# Impaired cortical mitochondrial function following TBI precedes behavioral changes

**DOI:** 10.3389/fnene.2013.00012

**Published:** 2014-02-04

**Authors:** William D. Watson, John E. Buonora, Angela M. Yarnell, Jessica J. Lucky, Michaela I. D’Acchille, David C. McMullen, Andrew G. Boston, Andrew V. Kuczmarski, William S. Kean, Ajay Verma, Neil E. Grunberg, Jeffrey T. Cole

**Affiliations:** ^1^Department of Neurology, Uniformed Services University of the Health SciencesBethesda, MD, USA; ^2^Department of Medical and Clinical Psychology, Uniformed Services University of the Health SciencesBethesda, MD, USA

**Keywords:** traumatic brain injury, energy metabolism, mitochondria, oxidative phosphorylation, animal behavior

## Abstract

Traumatic brain injury (TBI) pathophysiology can be attributed to either the immediate, primary physical injury, or the delayed, secondary injury which begins minutes to hours after the initial injury and can persist for several months or longer. Because these secondary cascades are delayed and last for a significant time period post-TBI, they are primary research targets for new therapeutics. To investigate changes in mitochondrial function after a brain injury, both the cortical impact site and ipsilateral hippocampus of adult male rats 7 and 17 days after a controlled cortical impact (CCI) injury were examined. State 3, state 4, and uncoupler-stimulated rates of oxygen consumption, respiratory control ratios (RCRs) were measured and membrane potential quantified, and all were significantly decreased in 7 day post-TBI cortical mitochondria. By contrast, hippocampal mitochondria at 7 days showed only non-significant decreases in rates of oxygen consumption and membrane potential. NADH oxidase activities measured in disrupted mitochondria were normal in both injured cortex and hippocampus at 7 days post-CCI. Respiratory and phosphorylation capacities at 17 days post-CCI were comparable to naïve animals for both cortical and hippocampus mitochondria. However, unlike oxidative phosphorylation, membrane potential of mitochondria in the cortical lining of the impact site did not recover at 17 days, suggesting that while diminished cortical membrane potential at 17 days does not adversely affect mitochondrial capacity to synthesize ATP, it may negatively impact other membrane potential-sensitive mitochondrial functions. Memory status, as assessed by a passive avoidance paradigm, was not significantly impaired until 17 days after injury. These results indicate pronounced disturbances in cortical mitochondrial function 7 days after CCI which precede the behavioral impairment observed at 17 days.

## INTRODUCTION

Traumatic brain injury (TBI) is a leading cause of death and disability in the United States, with an annual average of 53,014 deaths (Coronado et al., 2011). In developed countries, TBI is the most common cause of death or disability in children (Danielle van Pelt et al., 2011). Fortunately, these mortality rates appear to be declining, although this is primarily attributed to increasingly effective precautions such as airbags, improved child safety seats, and mandatory seat belt use (Coronado et al., 2011). To further reduce mortality and morbidity following TBI, increasing attention has been directed at putative secondary effects, including activated inflammatory responses, disrupted oxidative phosphorylation, and membrane potential, ROS production, calcium (Ca^2^^+^) dystasis, altering organelle ultrastructure and morphology, such as swelling and mitochondrial cristae deformation (He et al., 2004; Alves et al., 2005; Faria et al., 2007; Deshpande et al., 2008; Lloyd et al., 2008; Sun et al., 2008; Scafidi et al., 2009; Cole et al., 2010, 2011; Dalgard et al., 2012).

The brain is a highly aerobic, energy demanding tissue and as such is dependent upon mitochondria to maintain cerebral function. Post-traumatic perturbations in cellular bioenergetics and mitochondrial function have been documented by several researchers, and may trigger or exacerbate damaging secondary intracellular cascades after the primary injury. Altered glucose utilization (Hovda et al., 1991; Yoshino et al., 1991; Bergsneider et al., 1997, 2000, 2001; Moore et al., 2000; Casey et al., 2008; Scafidi et al., 2009), diminished energy production (Buczek et al., 2002; Marklund et al., 2006), functional mitochondrial changes (Sullivan et al., 1998, 2002; Harris et al., 2001; Lifshitz et al., 2003), and changes in key components of the aspartate malate shuttle (Casey et al., 2008; Scafidi et al., 2009; Cole et al., 2010), all provide potential mechanisms for rapid, exponential expansion of deleterious post-traumatic effects. Changes in energy generation and mitochondrial function are closely related to and interconnected with other delayed secondary manifestations of injury, including potent inflammatory responses (Dalgard et al., 2012) and Ca^2^^+^ dystasis (Ahmed et al., 2002; Weber et al., 2002; Louin et al., 2004; Deshpande et al., 2008; Balbino et al., 2010).

Given the extent of post-traumatic changes in mitochondrial function, and the possibilities of rapid amplification of secondary cascades, different therapies designed to minimize damage and retain/restore mitochondrial function after TBI are currently being actively studied. Neuroprotective treatments that attempt to delay or prevent secondary cascades include supplemental branched chain amino acids (BCAAs; Cole et al., 2010), cyclosporine A (Sullivan et al., 1999), low concentrations of uncouplers (Pandya et al., 2009), lactate (Ichai et al., 2009, 2013), peroxisome proliferator activated receptor gamma coactivator-1/peroxisome proliferator activated receptor agonists (Sauerbeck et al., 2011), pyruvate (Shen et al., 2010), fasting (Davis et al., 2008), ketogenic diets as well as ketone body administration (Prins et al., 2005; Appelberg et al., 2009). Early successes of these studies demonstrate the potential therapeutic value of pharmacological interventions which mediate mitochondrial protection in secondary brain injury.

However, despite pre-clinical successes, to date no treatment has successfully completed clinical trials. Therefore, to generate new treatments and refine therapies currently under development, perhaps a more detailed structure leading to a functional examination of the link between disruptions in mitochondrial function and behavioral (cognitive) impairment is required. While excellent work has investigated post-traumatic changes in energy metabolism and also cognitive deficits after injury, few studies have comparatively examined changes in cortical and hippocampal function while simultaneously quantifying changes in memory function. A more integrated approach looking at these two complementary brain regions will help broaden current understanding of post-traumatic brain bioenergetics.

## MATERIALS AND METHODS

### ANIMALS

All experiments were performed on male Sprague-Dawley rats, 12- to 16-weeks old, weighing ~350 g upon arrival (Taconic Farms, Hudson, NY, USA) and all procedures were approved by the Uniformed Services University of the Health Sciences (USUHS) Institution for Animal Care and Use Committee in accordance with international guidelines on the ethical use of animals. Overall wellness of the animals was assessed by monitoring weekly weights, water and feed consumption every other day. The living environment was thermostatically controlled at 21°C with 49% humidity in a reverse-light dark cycle facility with lights on at 1800 h and off at 0600. The animals were doubled-housed in standard rodent containers until the day of naïve treatment or surgical controlled cortical impact (CCI) at which time all animals were transferred to single housing for the remainder of the study. Standard rat chow (Teklad 18% protein rat chow, Harlan Teklad Diets, Madison, WI, USA) and fresh tap water were provided *ad libitum.*

### MODEL

Rats were randomly divided into four groups: 7 day naïve (*n* = 28) and CCI (*n* = 29), and 17 day naïve (*n* = 24) and CCI (*n* = 24). After 3 days of acclimation and gentling for behavioral analyses, all rats underwent treatment (naïve or injury). Both treatment groups were anesthetized with 4% isoflurane and 96% oxygen in an anesthetic chamber until loss of responsiveness to noxious stimuli. Anesthesia was maintained via nose cone administration of 2–3% isoflurane in 97–98% oxygen. All rats were then placed in a digital cranial stereotactic device and held in place by non-penetrating ear bars. Ophthalmic ointment was placed on their eyes, and a rectal probe was inserted no less than 5 cm; the probe was connected to a feedback warming blanket which maintained the blanket at 37°C. The naïve group received 2% isoflurane for 25 min followed by recovery. The injury group received a combined surgery/CCI brain injury. The surgical procedure of generating the craniotomy has been shown to cause significant injury responses across a spectrum of analytics (Cole et al., 2011). The CCI administration also causes injury responses in the rat brain. Therefore, this injury should be construed as a surgery/CCI injury. The cranium of the CCI group was then shaved. Craniotomies were performed -3.8 mm from bregma and centering 3.2 mm from the midline suture, with a 6.2 mm trephine to create a unilateral burr hole while avoiding the midline suture. This location was used in all procedures. TBI was administered by a CCI device (Benchmark^TM^ Stereotaxic Impactor) which delivered a 5 mm flat tip steel impactor to a depth of 2 mm at 5 m/s with a dwell time of 500 ms. The bone flap was replaced and held in position by bone wax (Ethicon) and the scalp incision was sutured.

### TISSUE COLLECTION

Rats were rendered unresponsive to toe-pinch by carbon dioxide (CO_2_) and euthanized by decapitation. Brains were quickly removed and placed on an iced operating platform. The cortex and hippocampus from the ipsilateral injury site were rapidly excised, placed in ice-cold buffer, and processed immediately for mitochondrial studies. Care was taken to collect cortical tissue in a consistent manner from the penumbra of injury and the corresponding region in the control brain. For subsequent measurement of enzyme activities and metabolite levels, cortex and hippocampus were flash-frozen in liquid nitrogen and processed as described below. For Western blots cortex and hippocampus were immediately homogenized after excision in RIPA buffer freshly supplemented with protease inhibitor cocktail for mammalian cells and tissues (Sigma P8340) and 100 μM EDTA.

For visual imaging of the lesion site, rats were anesthetized with sodium pentobarbital (50 mg/kg, i.p.) and perfused transcardially with phosphate buffered saline (pH 7.4) followed by 4% paraformaldehyde (pH 7.4, FD NeuroTechnologies, Inc., Baltimore, MD, USA). After dissection, the brain was post-fixed for one hour at 4°C and then transferred to 20% w/v sucrose in PBS solution for 2 days at 4°C. The brain was then sectioned via cryostat (Leica, Bannockburn, IL) into coronal cross sections (20 μm) and mounted onto chrome-alum-coated slides, which were stored in a -80°C freezer until analyzed.

### THIONIN STAINING

Slide sections of the brain were immersed in 0.1% Thionin (Aldrich, Milwaukee, WI, USA) for 4–6 s and washed in running water for 10 s followed by air drying. The slides were dipped in xylene for 3 s and then cover-slipped using Permount (Fisher Scientific, Hanover Park, IL, USA).

### POLAROGRAPHIC ANALYSIS OF OXIDATIVE PHOSPHORYLATION IN BRAIN HOMOGENATES

Immediately following tissue collection, 10% w/v homogenates were prepared in ice-cold buffer composed (in mM) of 210 mannitol, 70 sucrose, 10 HEPES, 1 EGTA, pH 7.2, freshly supplemented with +0.5% w/v fatty acid free-BSA and protease inhibitor cocktail for mammalian cells and tissues (Sigma P8340). Cortex and hippocampus were manually dispersed at 4°C using five to six strokes of a glass/Teflon homogenizer. Oxygen consumption was measured polarographically using a Strathkelvin oxygen electrode in a magnetically stirred, thermostatically regulated chamber (30°C). Aliquots of cortical and hippocampal homogenates were suspended in a total volume of 0.15 mL of air-saturated buffer composed (in mM) of 100 KCl, 75 mannitol, 25 sucrose, 5 KH_2_PO_4_, 0.05 EDTA, 1 EGTA, and 10 Tris pH 7.2. Oxygen consumption was measured in the presence of 10 mM pyruvate + 2 mM malate, 10 mM glutamate + 2 mM malate or 10 mM succinate + 2 μM rotenone followed by the addition of 0.15 mM ADP. State 3 is defined as the oxygen consumption following addition of substrate and 0.15 mM ADP, and may be referred to as “maximal induced oxygen consumption.” State 4 is the rate of oxygen consumption following depletion of exogenously added ADP. After achievement of state 4, uncoupler-stimulated oxygen consumption was measured by addition of 1 μM ClCCP. Cytochrome oxidase (complex IV) activity was measured using 2.5 mM ascorbate and 0.25 mM *N*,*N*,*N*′,*N*′,-tetramethyl-*p*-phenylenediamine (TMPD). Rates of substrate oxidation with or without ADP were expressed as natoms oxygen consumed/minute/mg protein. RCRs were calculated as the ratios of the rate of oxygen uptake in the presence of added ADP (state 3) to the rate observed when added ADP had been completely phosphorylated to ATP (state 4). Azide-sensitive NADH:O_2_ oxidoreductase activity was measured in the presence of 1 mM NADH and 25 μM horse heart cytochrome *c* after mitochondria diluted in 25 mM phosphate buffer, pH 7.2 were disrupted by freeze thawing. Deoxycholate (0.1% w/v) was added to assure complete permeabilization of the inner mitochondrial membrane. Protein concentration was determined by the BCA assay using BSA as standard.

### MITOCHONDRIAL MEMBRANE POTENTIAL

Using a similar method as that reported by Song et al. (2009), mitochondrial transmembrane potential was measured using tetramethylrhodamine, ethyl ester (TMRE) at excitation/emission wavelengths of 549 nm and 574 nm, respectively, in a Fluoromax-4 spectrofluorometer (Song et al., 2009). Aliquots of cortex and hippocampus homogenates were suspended in buffer composed (in mM) of 100 KCl, 75 mannitol, 25 sucrose, 10 Tris (pH 7.2), 5 KH_2_PO_4_, 0.05 EDTA, and 200 nM TMRE. Energization was achieved by addition of 10 mM pyruvate + 2 mM malate and depolarization by addition of 1 μM ClCCP. Membrane potential was calculated using the equation: MMP = [1 - (*F* + pyruvate + malate/*F* + ClCCP)] × 100, where *F* = fluorescence.

### MEASUREMENT OF ENZYME ACTIVITIES

Flash-frozen cortex and hippocampus from the ipsilateral injury site were used to prepare 10% w/v homogenates in ice-cold buffer composed (in mM) of 210 mannitol, 70 sucrose, 10 HEPES, 1 EGTA, and protease inhibitor cocktail for mammalian cells and tissues, at pH 7.8 (Sigma P8340). Enzyme assays were performed at 37°C using standard methods. Absorbance changes were continuously monitored using the dual-beam mode of an OLIS-converted DW2a spectrophotometer (OLIS, Bogart, GA). Specific activities are reported as nmol/min/mg protein. Citrate synthase was measured at 412 nm minus 360 nm (*ε* = 13.6 mM^-^^1^ cm^-^^1^) using 5,5-dithio-bis(2-nitrobenzoic acid) to detect free sulfhydryl groups in coenzyme A. Malate dehydrogenase (MDH) was measured by monitoring the decrease at 340 nm minus 450 nm (*ε* = 6.2 mM^-^^1^ cm^-^^1^) following oxidation of NADH. Protein concentration was determined by the BCA assay using BSA as standard.

### LACTATE AND PYRUVATE ANALYSES

Two different approaches were used to process tissue for lactate and pyruvate analysis. In the first approach, flash-frozen cortex and hippocampus were homogenized in a lysis buffer containing NP40 followed by rapid filtration through 10k MW cut-off microfuge filters to remove enzymes. In the second approach, flash-frozen cortex and hippocampus from the ipsilateral injury site were extracted at 4°C in 0.6 N HClO_4_. Protein pellets were solubilized in NaOH and protein concentration determined by the BCA assay using BSA as standard. Deproteinized extracts were neutralized to pH 6.5–7.0 with 3 M K_2_CO_3_/0.5 M triethanolamine. Lactate and pyruvate were determined in neutralized extracts and filtrates using Biovision assay kits according to manufacturer’s instructions.

### WESTERN BLOTS

After removal, cortex and hippocampus were immediately homogenized in RIPA buffer freshly supplemented with protease inhibitor cocktail for mammalian cells and tissues (Sigma P8340) and 100 μM EDTA. Aliquots of RIPA lysates were frozen at -80°C prior to analysis and protein was quantified with the BCA assay using BSA as standard. Aliquots of RIPA lysates were diluted in Laemmli sample buffer and boiled for 2 min. Equivalent amounts of protein (40 mg) were loaded on 4–12% Tris–glycine gradient gels (Invitrogen, Carlsbad, CA, USA) and subsequently transferred to PVDF membrane (Invitrogen) at 4°C. After transfer to PVDF, membranes were blocked with 2.5% non-fat dairy milk (NFDM) in PBS for 1 h. Membranes were analyzed with the following antibodies: pyruvate carboxylase (Santa Cruz Biotechnology; 1/750); pyruvate dehydrogenase (PDH) E1-α (Invitrogen; 1/1000); pyruvate kinase (Abcam; 1/1000), glutamate dehydrogenase (GenWay; 1/1000); and citrate synthase (Abcam; 1/750). Incubation with mouse polyclonal β-actin (Sigma; 1:10,000; 24 h), was used as a loading control. After three 5 min washes with TBS-T, membranes were incubated with species appropriate anti-goat (1:5000) from Santa Cruz, anti-mouse (1:5000) and anti-rabbit (1:5000) from Cell Signaling secondary antibodies for 1 h. Enhanced chemiluminescence detection (Millipore, Billerica, MA, USA) was used to detect the bands. After collection of the images with a FluorChem HD2 camera (Cell Biosciences, Santa Clara, CA, USA), Alpha Ease FC densitometry analysis software (Cell Biosciences) was used to quantify protein expression levels between treatments and normalized to β-actin for later statistical analysis.

### PASSIVE AVOIDANCE BEHAVIORAL TESTING

Memory was assessed using a passive avoidance paradigm, in which rats were trained and tested in two Med Associates shuttle boxes (EVN-018MD, St. Albans, VT, USA). The apparatus consists of two boxes (21 cm × 25 cm × 17 cm each) separated by a mechanical door. On the training day a rat was placed in the illuminated box (50 W bulb). After 1 min, the door between the boxes opened. If the rat crossed into the dark box, the door closed and a 0.8 mA shock was administered through the metal grid floor for 5 s. The rat was tested 24 h later and its latency to cross into the dark box was measured. An increase in the amount of time (in seconds) to cross into the dark chamber reflects memory of the shock. Latency measurements (no shock administered) were taken six times during this experiment: baseline, and two days after injury [2 days, post injury day (PID)], followed by additional measures 7 PID, and 17 PID after injury. If the rat did not cross into the dark box, the test was stopped after 5 min.

### STATISTICAL ANALYSIS

Collected data were analyzed via ANOVA using SigmaStat (Systat Software, Chicago, IL, USA). When normality tests failed, a Kruskal–Wallis ANOVA on ranks was performed. Significance was based on two-tailed tests, with *p* < 0.05. For post hoc comparisons, Holm–Sidak tests were used. Data analysis was performed at the conclusion of the project, after all results were collected.

## RESULTS

### CONTROLLED CORTICAL IMPACT CAUSED A CORTICAL LESION DIRECTLY ABOVE THE HIPPOCAMPUS

**FIGURE 1:**
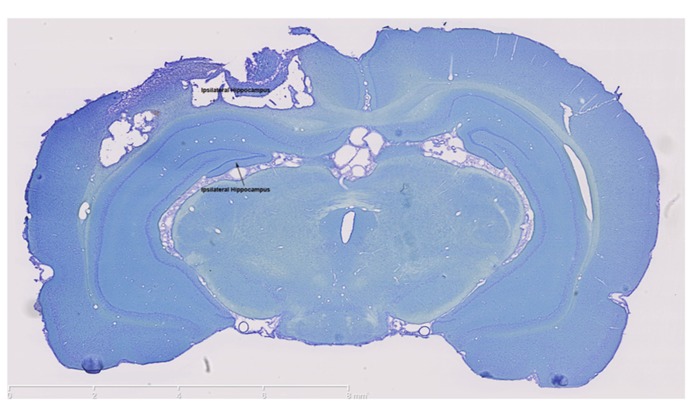
**The controlled cortical impact model causes a large lesion in the ipsilateral cortex.** Thionin staining clearly shows extensive damage in both the ipsilateral cortex and ipsilateral hippocampus which were the brain regions targeted for injury. In contrast, thionin staining demonstrates an intact contralateral cortex and hippocampus. Scale bars are show on each image, which are representative images from a collection of *n* = 3 for control and injured rats.

The impact of the CCI plunger caused a large lesion directly above the hippocampus (**Figure 1**). The lesion served as the focal point for the tissue collected in the injured animals (as well as the identical anatomical region in the control group) for experimental analysis. The clear damage to the cortex in this region, and the shifting of the ipsilateral hippocampus were used in part to select the behavioral tests.

### TRAUMATIC BRAIN INJURY CAUSES IMPAIRED MITOCHONDRIAL FUNCTION IN THE CORTEX

**FIGURE 2:**
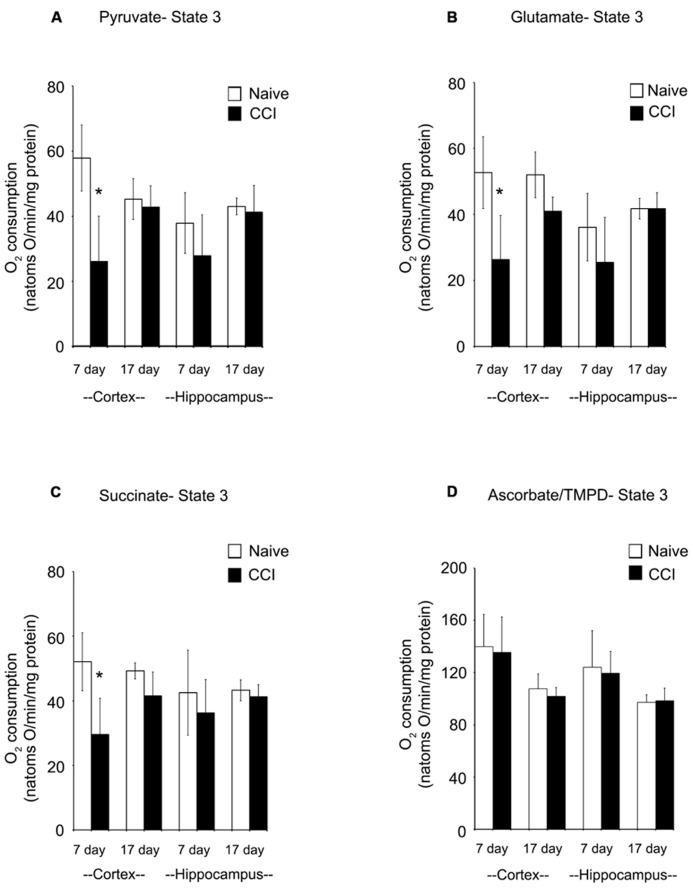
**In the cortex, traumatic brain injury significantly reduces state 3 oxidative phosphorylation.** The addition of pyruvate **(A)** and glutamate **(B)** to cortical and hippocampal homogenates revealed a significant impairment in complex 1 of the electron transport chain of the cortex 7 days after an injury. After 17 days, this deficiency was no longer observable. Succinate **(C)** addition demonstrated a similar impairment pattern in complex 2, with only the cortex affected 7 days after an injury. Complex 4 was not affected by injury, as seen following addition of ascorbate/TMPD **(D)**. At no timepoint after injury was the hippocampus affected by injury. Asterisk denotes a significant difference when compared to the control group, with significance declared at *p* < 0.05. All values are means ± SD (*n* = 12).

In cortical mitochondria, state 3 respiration following activation of complex I of the electron transport chain by addition of pyruvate + malate or glutamate + malate or activation of complex II by addition of succinate was significantly decreased (*p* < 0.05) 7 days after CCI compared to corresponding naïve rats (**Figures 2A–C**). By 17 days after CCI, state 3 respiration had returned to normal in the cortex. In the hippocampus, respiratory rates did not differ (*p* > 0.05) between groups for day 7 or 17 ipsilateral hippocampus, demonstrating that while mitochondrial function was markedly impaired in the cortical injury site, the hippocampus was largely spared. Addition of TMPD + ascorbate (**Figure 2D**), which activates complex IV did not reveal a significant change in oxidative phosphorylation capacity in either brain region at either timepoint tested.

### BRAIN INJURY CAUSES IMPAIRED MITOCHONDRIAL FUNCTIONAL INTEGRITY

**FIGURE 3:**
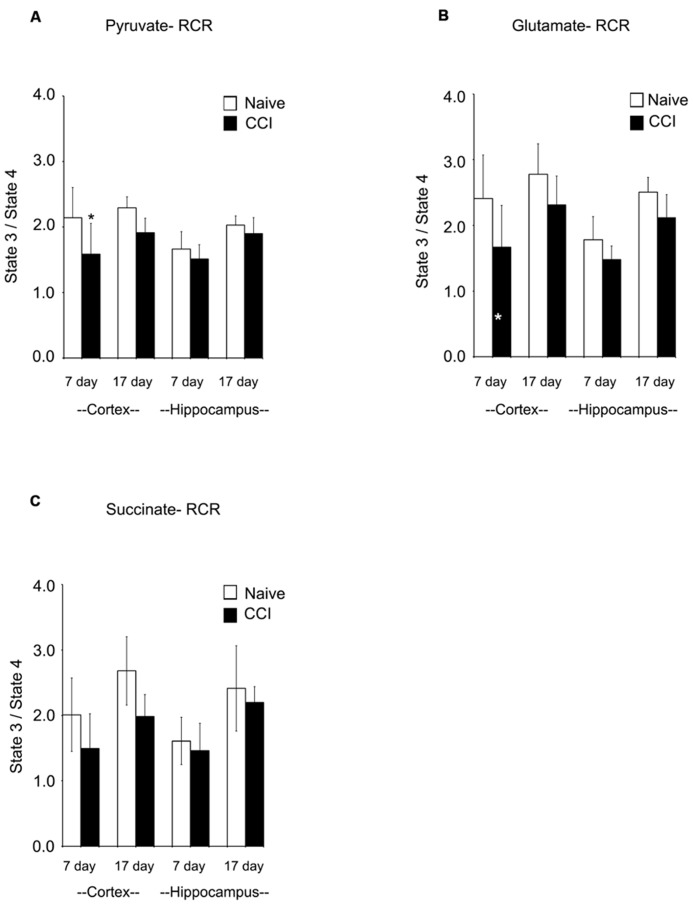
**Following traumatic brain injury, cortical respiratory control ratio (RCR), an indicator of mitochondrial health, is significantly impaired.** The addition of both pyruvate **(A)** and glutamate **(B)** reveal significant impairments in the RCR in the cortex 7 days after injury. The addition of succinate **(C)** did not demonstrate a deficiency in RCR. At no time was hippocampal RCR effected by injury. Asterisk denotes a significant difference when compared to the control group, with significance declared at *p* < 0.05. All values are means ± SD (*n* = 12).

Respiratory control ratios (RCRs) were calculated as the ratio of the rate of oxygen uptake in the presence of added ADP (state 3) to the rate observed when added ADP had been completely phosphorylated to ATP (state 4). The RCR reflects the degree of coupling between substrate oxidation and ADP phosphorylation and is a commonly used indicator of the physical and functional integrity of mitochondria. RCRs following pyruvate or glutamate addition (**Figures 3A,B**) were significantly decreased (*p* < 0.05) 7 days after CCI in cortical mitochondria but not after 17 days (*p* > 0.05); following succinate addition (**Figure 3C**) to 7 day cortical mitochondria, the post-traumatic changes in RCR approached but did not attain a statistically significant decrease (*p* = 0.071). Decreases in RCR in 7 day cortical mitochondria resulted from a decrease in state 3 rather than an increase in state 4, suggesting that endogenous proton conductance of the inner mitochondrial membrane was not significantly affected. In 7 and 17 day ipsilateral CCI hippocampal mitochondria, RCR values were similar to naïve animals. In sum, these observations show that respiratory and phosphorylation activities of cortical mitochondria present in the impact area are severely injured while those in the ipsilateral hippocampus at 7 days post-CCI are essentially normal.

**FIGURE 4:**
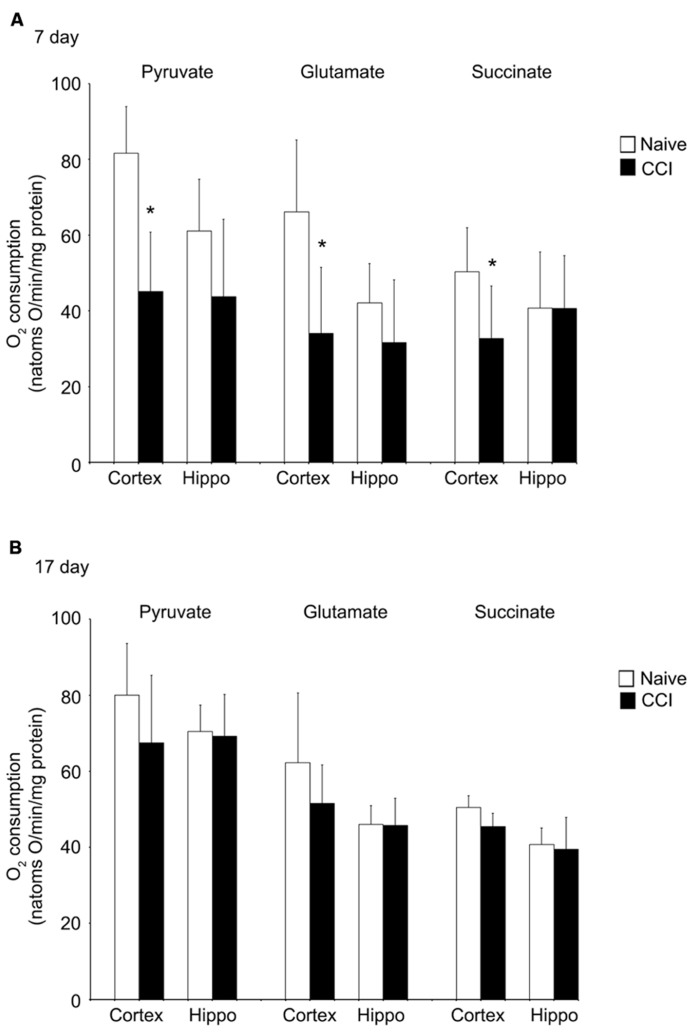
**Following traumatic brain injury, uncoupler-stimulated (maximal) oxygen consumption is significantly impaired in the cortex.** In the cortex, but not the hippocampus, mitochondrial membrane potential **(A)** is significantly reduced 7 days after an injury. No difference was observed at 17 days post-injury **(B)**. In neither brain region was NADH oxidase affected by injury. *Denotes a significant difference when compared to the control group, with significance declared at *p* < 0.05. All values are means ± SD (*n* = 12).

Uncoupled respiration is controlled by substrate oxidation, which is a cumulative result of substrate transport, dehydrogenase activity and the electron transport chain. After state 4 respiration had been achieved, uncoupler-stimulated (maximal) oxygen consumption was measured by addition of ClCCP. In naïve samples, uncoupler moderately stimulated oxygen consumption above the state 3 rate elicited by addition of ADP with pyruvate and glutamate (but not with succinate). However, the magnitude of increase, as seen in **Figures 4A,B**, was significantly decreased in day 7 cortical mitochondria incubated with pyruvate, glutamate, or succinate. In hippocampal tissue, uncoupled oxygen consumption in response to substrate addition was not affected (*p* > 0.05). Uncoupled respiration in both injured cortex and hippocampus was similar to naïve animals at day 17. The observation that uncoupler does not significantly elevate oxygen consumption above that stimulated by ADP suggests that decreased rates of state 3 rates of respiration seen in cortical mitochondria at day 7 do not result from restricted proton re-entry through the ATP synthase but rather suggest that the defect in oxidative phosphorylation observed in day 7 CCI cortical mitochondria is located on the oxidative side of the electron transport chain.

### MITOCHONDRIAL FUNCTION IS IMPAIRED BY TRAUMATIC BRAIN INJURY

**FIGURE 5:**
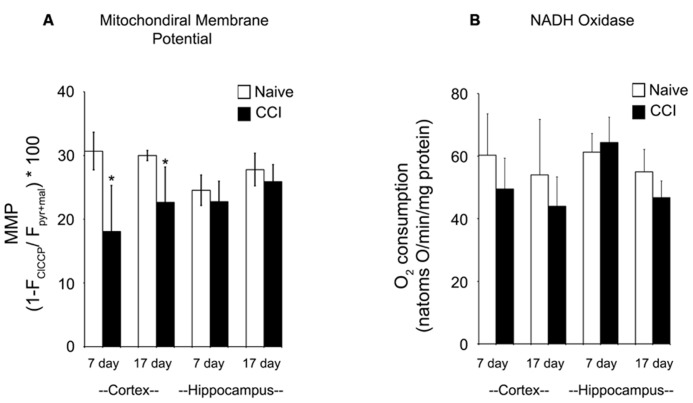
**Mitochondrial membrane potential generation is significantly impaired in the cortex after a brain injury.** At both 7 and 17 days after injury, mitochondrial membrane potential **(A)** is significantly reduced in the cortex, but not the hippocampus. In contrast, at neither timepoint in both the cortex and the hippocampus is NADH oxidase activity **(B)** altered by injury. Asterisk denotes a significant difference when compared to the control group, with significance declared at *p* < 0.05. All values are means ± SD (*n* = 12).

The maximum transmembrane potential that brain mitochondria could generate was measured using TMRE to differentiate between mitochondria fully energized with pyruvate + malate and fully depolarized by addition of the uncoupler ClCCP. Membrane potential is a key indicator of inner membrane integrity and the bioenergetic status of mitochondria. As shown in **Figure 5A**, cortical mitochondria exhibited a significant decrease in membrane potential at 7 days relative to the corresponding naïve animals (*p* < 0.01) and a smaller but still significant decrease at 17 days post-CCI compared to naïve animals (*p* < 0.05). By contrast, hippocampal mitochondrial membrane potential was comparable to corresponding naïve animals at both 7 and 17 days post-CCI (*p* > 0.05). The observation that membrane potential was decreased at 17 days cortical tissue in CCI-injured animals but oxidative phosphorylation was not significantly affected may have important long-term implications for other membrane potential-sensitive functions of mitochondria, including Ca^2^^+^ sequestration and ROS formation.

Given the significant impairments in oxidative phosphorylation and mitochondrial membrane potential after an injury, azide-sensitive NADH:O_2_ oxidoreductase (NADH oxidase) activity (**Figure 5B**) was quantified in disrupted mitochondria in the presence of exogenous NADH to assure full accessibility of NADH dehydrogenase in complex I to NADH. NADH oxidase was comparable in naïve and 7 and 17 day CCI mitochondria, demonstrating that overall capacity of and electron flow through the electron transport chain were not impaired in CCI cortical and hippocampus mitochondria at either time point. These observations indicate that the energetic defect observed in 7 day CCI cortical mitochondria reside can be attributed to a CCI-mediate rate limitation of reactions supplying NADH to the electron transport chain, combined with impaired function of complexes I or II.

### EXPRESSION OF KEY PROTEINS NOT AFFECTED BY BRAIN INJURY

**FIGURE 6:**
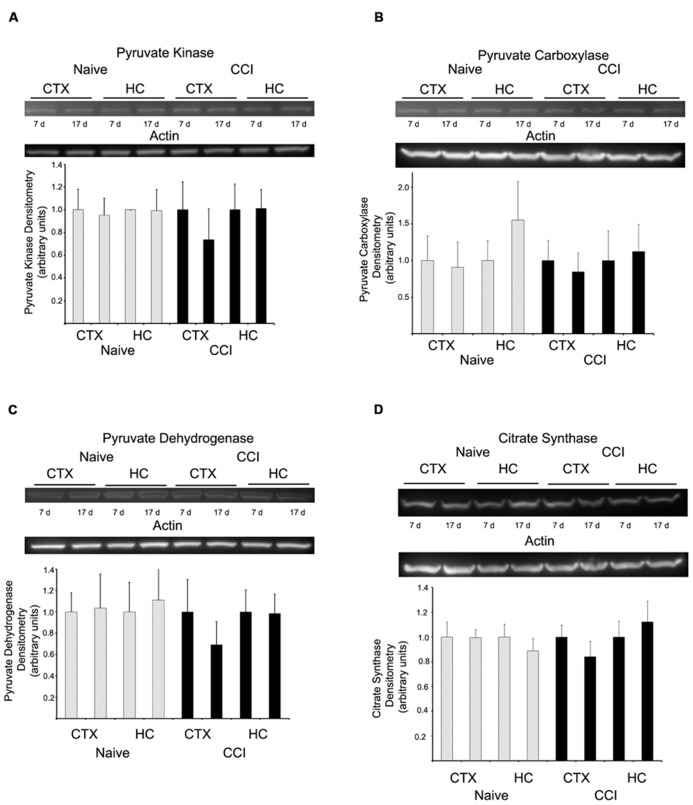
**Western blot analysis revealed no change in the expression of key metabolizing proteins.** In both hippocampus and cortex, at neither 7 nor 17 days after injury were pyruvate kinase **(A)**, pyruvate carboxylase **(B)**, pyruvate dehydrogenase **(C)**, or citrate synthase **(D)** altered. Blots are representative of an *n* = 4 for each protein, with each blot loaded with one of each sample type. Densitometry is presented as means ± SD.

Western blots were performed to determine if substrate oxidation in day 7 cortical mitochondria might be limited by decreased amounts of metabolic enzymes, in particular those involved in pyruvate utilization, the substrate which appeared most markedly affected by CCI at this time point. There was no difference in the level of pyruvate kinase, pyruvate carboxylase, PDH E1-α, or citrate synthase (**Figure 6**), at either 7 or 17 days in cortical or hippocampus regions nor was there a decrease in glutamate dehydrogenase (data not shown).

The accumulated results suggest that the defect in state 3 respiration observed in day 7 CCI-injured mitochondria precedes the electron transport chain and resides at the level of either substrate transport into the matrix or of substrate oxidation. However, these effects appear to be diminished or absent by 17 days after injury. Therefore, the activity of MDH or citrate synthase was investigated, since these enzymes are vital precursors in the generation of energy.

Measurement of citrate synthase demonstrated decreased activity at 7 days in cortical mitochondria (**Table 1**), but not at 17 days. In contrast, total MDH (cytosolic + mitochondrial) in day 7 and 17 day cortex and hippocampus homogenates showed that MDH activity was comparable in both naïve and CCI brain at 7 and 17 days (**Table 1**). The reaction catalyzed by MDH which converts malate to oxaloacetate (OAA) in the Krebs cycle is strongly endergonic with the equilibrium of this reaction favoring the formation of malate. However, *in vivo* the MDH reaction is pulled in the forward direction by the highly exergonic reaction catalyzed by citrate synthase which rapidly removes OAA, thus decreasing the matrix concentration and pulling the MDH reaction toward formation of OAA. These results suggest that pyruvate oxidation may be negatively impacted by a decrease in citrate synthase in day 7 cortical mitochondria by limiting condensation of acetyl CoA generated in the PDH reaction with OAA in the reaction catalyzed by citrate synthase. Malate is added as a co-substrate when pyruvate oxidation is measured in mammalian mitochondria to assure that acetyl CoA generated by PDH is rapidly removed by citrate synthase. A slowed rate of citrate synthase would be expected to elevate the ratio of acetyl CoA/CoA which in turn would allosterically inhibit pyruvate oxidation by PDH.

**Table 1:** Cortical citrate synthase activity is significantly decreased 7 days after traumatic brain injury.

	Cortex		Hippocampus	
	Naïve	CCI	Naïve	CCI
Citrate synthase	(nmol/min/mg protein)		(nmol/min/mg protein)	
7 days after injury	410 ± 35	317 ± 76*	466 ± 59	431 ± 16
17 days after injury	458 ± 39	426 ± 21	494 ± 84	440 ± 61
Malate dehydrogenase	(μmol/min/mg protein)		(μmol/min/mg protein)	
7 days after injury	4.62 ± 0.61	4.06 ± 0.99	4.99 ± 0.85	5.12 ± 0.89
17 days after injury	4.26 ± 0.34	4.24 ± 0.58	4.36 ± 0.48	3.91 ± 0.46

*Denotes a significant difference in comparison to control, *p* < 0.05. All values are means ± SD, with a *n* = 12.

### LACTATE/PYRUVATE RATIO NOT AFFECTED BY INJURY

A decreased rate of pyruvate oxidation by mitochondria and/or increased rate of glycolysis would be predicted to elevate the lactate/pyruvate ratio. However, in neither the cortex nor the hippocampus was there a significant increase (*p* > 0.05) in the lactate/pyruvate ratio at either 7 or 17 days after CCI (**Table 2**). Current evidence suggests a range of outcomes indicative of increased anaerobic/decreased aerobic metabolism after TBI, including an increase in the lactate:pyruvate ratio, increases in lactate, or increases in both lactate and pyruvate with no change in the lactate/pyruvate ratio. There is considerable variability inherent in the methods used to quantify these parameters, as well as considerable differences in the conditions under which the data were collected (e.g., time and severity of injury, tissue collection and analysis, chemical instability of pyruvate). Indeed, there is significant variability between our two sets of metabolite determinations. Despite the inability to document an elevated lactate/pyruvate ratio, it is clear that TBI disrupts bioenergetic function of mitochondria in injured cortex at 7 days post-CCI.

**Table 2:** In neither brain region tested after an injury, at either timepoint, was the lactate:pyruvate ratio altered.

Lactate/pyruvate ratio	Cortex		Hippocampus	
	Naïve	CCI	Naïve	CCI
	L/P		L/P	
7 days after injury	12.23 ± 1.30	15.33 ± 5.33	7.49 ± 0.33	8.46 ± 1.71
17 days after injury	13.13 ± 0.63	12.02 ± 3.78	5.71 ± 2.50	5.49 ± 1.78

### MITOCHONDRIAL DEFICITS PRECEDE ALTERATIONS IN BEHAVIORAL RESPONSES

**FIGURE 7:**
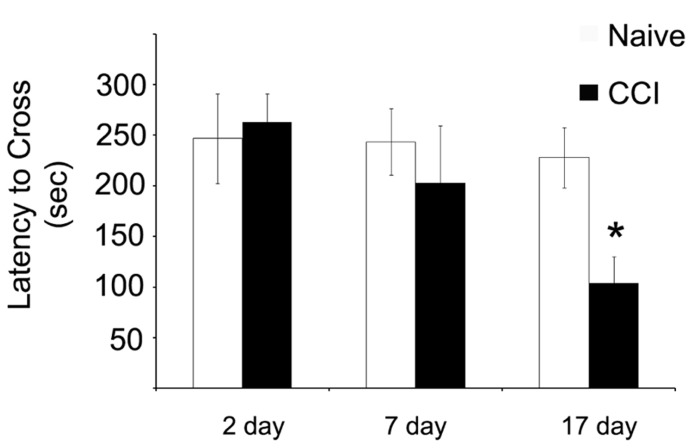
**Memory function as measured by a passive avoidance paradigm is impaired after traumatic brain injury, but this phenomenon does not occur until after significant impairments in mitochondrial function.** When animals were given aversive stimuli the day after the injury and tested for their ability to remember stimuli 2, 7, and 17 days after the injury, CCI rats were significantly impaired in recall ability. Asterisk denotes a significant difference when compared to the control group, with significance declared at *p* < 0.05. All values are means ± SD (*n* = 12).

Having demonstrated significant impairments in mitochondrial bioenergetic capacity, the effect of TBI on aversive stimuli avoidance task was quantified. Using a passive avoidance paradigm, in which rats were trained the day after the injury and then tested 2, 7, and 17 days after CCI, the length of time the rats avoided a chamber in which they previously received an aversive stimuli (mild footshock) was recorded (**Figure 7**). No effect of injury was observed 2 days after injury. However, it appeared that there was a trend toward a significant decrease in the avoidance time in the rats when tested 7 days after injury, although this did not reach significance. Most interestingly, however, the performance of the injured rats continued to decline until by 17 days after injury, in which case they would enter the avoidance chamber significantly faster than would the naïve animals, indicating a loss of memory of the previous aversive stimuli.

## DISCUSSION

The key result of this study is that significant impairments in cellular bioenergetic capacity precede alterations in behavioral performance. Using homogenized cortical and hippocampal tissue to preserve potentially damaged and fragile mitochondria, oxidative phosphorylation assays demonstrated a profound disruption in the electron transport chain. However, this impairment in electron transport chain function was limited to complex 1 and 2, with further possible disruptions occurring prior to substrate entry into the ETC, with no difference observed in complexes 3 or 4. This result, a partial disruption in ETC function, may be suggestive of an overriding mechanism. This laboratory previously demonstrated significant reductions in both isoforms of aspartate aminotransferase (AAT-1 and ATT-2), as well as significant increases in glutamate dehydrogenase (Cole et al., 2010). These enzymes, key to the proper function of the TCA cycle, also play a vital role in the aspartate–malate shuttle. Disruption of either biochemical process will have tremendous, far-reaching effects. Disruption of the TCA cycle, either via diminished provision of OAA via aspartate, or removal of glutamate by GDH will obviously reduce the number of reducing equivalents being generated for entry into the TCA cycle. Alternatively, disruption of the equilibration of NAD^+^/NADH across the mitochondrial membrane, a key function of the aspartate–malate shuttle, may ultimately disrupt the capacity of the ETC to translocate protons across the mitochondrial membranes.

In addition to changes in protein expression, recent reports show significant changes in post-traumatic protein phosphorylation, indicative of altered enzyme activity. PDH has previously been observed to have an altered phosphorylation status after injury (Xing et al., 2009). Those results indicate that 7 days after brain injury, there is a significant post-traumatic decrease in the ratio of phosphorylated PDH:PDH. This finding is intriguing, as the phosphorylated form of the PDH is inactive. Put another way, there is a relative increase in the percentage of active PDH 7 days after a brain injury. This is intriguing since PDH activity would be expected to increase the conversion of pyruvate to acetyl CoA for entry into the TCA cycle, with a concomitant decrease in lactate. This could possibly be a compensatory response to overcome deficiencies in pre-electron transport chain mechanisms.

Given this pre-existing data, perhaps the most unexpected finding was the lack of changes in lactate, pyruvate, and lactate:pyruvate (L:P) ratio. However, given the ambiguity in previously published work monitoring post-traumatic changes in these parameters, our result is perhaps not surprising (Nelson et al., 2004; Vespa et al., 2007a,b; Hutchinson et al., 2009; Zygun et al., 2009). This, to our knowledge, is one of the few studies to actually remove the injured region of the brain and quantify shifts in lactate and pyruvate and then calculate the ratio of the two. Multiple explanations exist for lack of change in these parameters. Possibly, the absence of significant changes occurred because of the collection of tissue 7 days after the TBI was administered, while earlier collection of the tissue, within hours of injury, may have detected differences in the L:P ratio. In this case, a shift to anaerobic metabolism may be precipitated by a transient hypoxic/ischemic state induced by the injury. Some evidence suggests regionally specific changes in microvasculature can persist for at least 2 weeks after injury. However, the initial diminution of oxygen to the injured region is likely restored relatively quickly after a TBI. This in turn, would lead to a restoration of aerobic metabolism as the primary means of generating ATP. Therefore, any putative regionally specific shifts in long-term (i.e., days to weeks) brain bioenergetics would likely be done via altered protein levels or activity and not hypoxia/ischemia. This concept is supported by previous work from this laboratory (Cole et al., 2010). Following a TBI, the aspartate–malate shuttle is profoundly impaired. While the full ramifications of this phenomenon have not yet been elucidated, one key effect will be a disruption in the balance of reducing equivalents NAD^+^/NADH across the mitochondrial membrane. This imbalance will greatly reduce the capacity of the mitochondria to metabolize TCA cycle products and result in both decrease aerobic metabolic cycle rates, but indirectly increase the conversion of pyruvate to lactate. However, the far-reaching results of a change in the aspartate–malate shuttle will further impair the entire process of glycolysis and TCA cycle metabolism, so while the conversion of pyruvate to lactate is increased, the net production of both metabolites may be slightly reduced due to limited glycolytic activity. This makes interpretation of the lactate:pyruvate data problematic in any study.

These results suggest that cognitive impairment is preceded by disruptions in the metabolic and bioenergetic capacity of the injured regions of the brain. When these results are coupled with those indicating changes in cytokine profiles (Dalgard et al., 2012), amino acid metabolism (Cole et al., 2010), others (Hovda et al., 1990, 1991; Bergsneider et al., 2000, 2001; Bartnik et al., 2007; Casey et al., 2008), there appears to be a large multi-factorial response that culminates in the clinical manifestations of injury. However, the sequence and interrelated causality of these events has not been determined. Given the central nature of cellular bioenergetics, it can be inferred that diminished energy generating capacity, and a shift from aerobic to anaerobic metabolism would be a key player in escalating the secondary cascade of events. Therefore, early intervention limiting the extent of changes in mitochondrial disruption, coupled with treatments that ameliorate other observed changes in brain function including anti-inflammatory treatments, nutriceutical intervention and manipulation of bioenergetic substrates may provide the best option for limiting or preventing cognitive deficits after injury.

Further work will need to be done to evaluate the exact timecourse of metabolic changes in the brain after TBI. Additional data regarding other post-traumatic alterations in the brain, such as potent cytokine responses, will be needed to determine the optimal intervention to limit changes in energy generation, and ultimately cognitive function. While certainly some treatments can be applied within hours of injury, there is a large segment of the TBI population that would perhaps not even seek medical care within hours of an injury. More specifically, male adolescents, who are both more prone to injury and less likely to seek immediate care, would likely arrive at a care center after an initial “storm of events” has occurred. By understanding the secondary cascades that perhaps arise from these immediately post-injury events that occur within hours of injury, treatment can be developed to best target the secondary cascade of events.

The direct causal relationship between mitochondrial dysfunction and behavioral impairment is strictly correlative at this time. While several of the assays demonstrated a return to control values 17 days after injury, this should not be interpreted as a complete restoration of mitochondrial function. Indeed, mitochondrial membrane potential, which is a measure of the culmination of a complex series of subcellular events including transmembrane transport function, electron transport chain activity, and enzymatic activity, continued to be impaired 17 days after injury to the cortex. It may be that assays that look too closely at a subcellular function and observe no major differences miss larger scale changes that are the culmination of small, inconclusive alterations. It is likely that the membrane potential is just such an assay. It should also be noted that a single behavioral task was used to examine cognitive function, and motor function was not evaluated. Additional analyses evaluating cognitive performance more extensively, including tests of anxiety, depression and additional variants of learning and memory tasks, would allow the refinement of the linkage between energy metabolism and behavioral impairment. It should be noted that the passive avoidance task is thought to be primarily hippocampal (and amygdala) dependent, with some peripheral cortical involvement. The fact that impairment is observed in the passive avoidance assay despite the relative good health of the hippocampus is explainable by the significant impairments that persist in the cortex. This interesting result only heightens the need for further exploring the impact of mitochondrial dysfunction in both hippocampus and cortex on memory. By supplementing behavioral tests with experiments evaluating motor ability such as beam walking/beam balancing assessments, as well as a wider variety of cognitive tasks such as object recognition, significant insight could be gained into which brain regions are affected as well as possible mechanisms. Future investigation of this topic attempting to tie mitochondrial dysfunction to a neurological outcome will be challenging. Given that TBI alters such a wide range of cellular processes, research examining mitochondrial function should be aware of the host of other related pathways that may contribute equally, if not more, to neurological deficits. One area of key importance will be the possible regulatory effects of inflammatory responses after injury. Recent work suggests that within hours of injury, if not minutes, there is a very potent inflammatory response. Cytokine expression remained elevated for several days after injury, and in some cases this elevation persisted for more than a week (Dalgard et al., 2012). These inflammatory responses could in fact be the key initiator of the “secondary cascade of events” that occurs after a TBI and ultimately culminates in neurological deficits. Additionally, future studies would benefit from using additional behavioral paradigms. Specifically, tasks that quantify changes in motor function as well as indicators of anxiety would be essential to fully understanding the role of bioenergetics dysfunction and behavioral deficits.

## DISCLAIMER

The opinions expressed herein belong solely to the authors. They do not nor should they be interpreted as representative of or endorsed by the Uniformed Services University of the Health Sciences, U.S. Army, U.S. Navy, Department of Defense, or any other agency of the federal government.

## Conflict of Interest Statement

The authors declare that the research was conducted in the absence of any commercial or financial relationships that could be construed as a potential conflict of interest.
